# Dickkopf-3 Upregulates VEGF in Cultured Human Endothelial Cells by Activating Activin Receptor-Like Kinase 1 (ALK1) Pathway

**DOI:** 10.3389/fphar.2017.00111

**Published:** 2017-03-14

**Authors:** Carla L. Busceti, Simona Marchitti, Franca Bianchi, Paola Di Pietro, Barbara Riozzi, Rosita Stanzione, Milena Cannella, Giuseppe Battaglia, Valeria Bruno, Massimo Volpe, Francesco Fornai, Ferdinando Nicoletti, Speranza Rubattu

**Affiliations:** ^1^IRCCS NEUROMED - Istituto Neurologico MediterraneoPozzilli, Italy; ^2^Department of Physiology and Pharmacology, Sapienza University of RomeRome, Italy; ^3^Department of Clinical and Molecular Medicine, Sapienza University of RomeRome, Italy; ^4^Department of Human Morphology and Applied Biology, University of PisaPisa, Italy

**Keywords:** Dickkopf-3, TGF-β1, ALK1, VEGF, angiogenesis

## Abstract

Dkk-3 is a member of the dickkopf protein family of secreted inhibitors of the Wnt pathway, which has been shown to enhance angiogenesis. The mechanism underlying this effect is currently unknown. Here, we used cultured HUVECs to study the involvement of the TGF-β and VEGF on the angiogenic effect of Dkk-3. Addition of hrDkk-3 peptide (1 or 10 ng/ml) to HUVECs for 6 or 12 h enhanced the intracellular and extracellular VEGF protein levels, as assessed by RTPCR, immunoblotting, immunocytochemistry and ELISA. The increase in the extracellular VEGF levels was associated to the VEGFR2 activation. Pharmacological blockade of VEGFR2 abrogated Dkk-3-induced endothelial cell tubes formation, indicating that VEGF is a molecular player of the angiogenic effects of Dkk-3. Moreover, Dkk-3 enhanced Smad1/5/8 phosphorylation and recruited Smad4 to the VEGF gene promoter, suggesting that Dkk-3 activated ALK1 receptor leading to a transcriptional activation of VEGF. This mechanism was instrumental to the increased VEGF expression and endothelial cell tubes formation mediated by Dkk-3, because both effects were abolished by siRNA-mediated ALK1 knockdown. In summary, we have found that Dkk-3 activates ALK1 to stimulate VEGF production and induce angiogenesis in HUVECs.

## Introduction

Dickkopf-3 (Dkk-3) is a divergent member of the Dkk protein family of secreted inhibitors of the Wnt pathway, which exerts pleiotropic functions in different biological contexts (Niehrs, 2006). Unlike Dkk-1 and other Dkk family members, a precise role for Dkk-3 in the modulation of the Wnt pathway has not been identified to date (Krupnik et al., 1999; Wu et al., 2000; Mao B. et al., 2001; Mao J. et al., 2001; Mao et al., 2002; Diep et al., 2004; Veeck and Dahl, 2012).

Of note, human Dkk-3 was proposed to function as a tumor suppressor because its expression was shown to be down-regulated in cancer cells (Nozaki et al., 2001; Kurose et al., 2004; Lodygin et al., 2005; Kuphal et al., 2006). Dkk-3 might also display angiogenic activity during embriogenesis and tumor development (Untergasser et al., 2008). The molecular mechanisms underlying the angiogenic activity of Dkk-3 are currently unknown. Studies carried out in tumor cell lines have shown that Dkk-3 enhanced phosphorylation of Smad3 (Kashiwakura et al., 2008), a molecule that lies along the signaling pathway activated by TGF-β1. The latter has an established role in angiogenesis (Lebrin et al., 2005; López-Novoa and Bernabeu, 2010).

TGF-β1 acts as a modulator of endothelial cell proliferation and migration by activating two subtypes of TGF-β1 type I receptors named ALK5 (Activin like kinase 5) and ALK1 (Activin like kinase 5). In particular, TGF-β1 enhances endothelial cell migration and proliferation by activating the ALK1 receptor subtype whereas it does the opposite acting on ALK5 receptor (Goumans et al., 2002; Lebrin et al., 2005). TGF-β can modulate angiogenesis by regulating the expression of VEGF in vascular endothelial cells (Seghezzi et al., 1998; Ferrara et al., 2003; Ferrara, 2004; Ferrari et al., 2006). Of note, VEGF is under the transcriptional control of TGFβ-/Smad signaling pathway (Clifford et al., 2008).

The direct evidence that Dkk-3 activates the TGF-β/VEGF pro-angiogenic pathway is still lacking.

The aim of our study was to characterize the molecular mechanism underlying the angiogenic activity of Dkk-3 by assessing the involvement of the TGF-β1 intracellular signaling and testing the hypothesis that VEGF may be a transcriptional target of Dkk-3 in HUVECs.

## Materials and methods

### Materials

hrDkk-3 peptide (R&D Systems, Minneapolis, MN, USA); ML347 (Tocris Bioscience, Bristol, UK); Oligo: SASI_Hs01_00113768 (Sigma Aldrich, Milan, Italy); Recombinant Human TGF beta-1 (Millipore, Billerica, MA, USA); ZM 323881 (Tocris Bioscience).

### Endothelial cell cultures

Commercially available HUVECs (Lonza, Walkersville, MD, USA, code: CC-2517, lot number: 0000323352) were used within 4 passages for *in vitro* experiments. These cells have been reviewed by Quality Assurance in compliance with requirements of Lonza's Quality System. Cells were cultured in endothelial growth medium-2 (EGM-2) (Lonza) supplemented with 10% dialyzed fetal bovine serum (Life Technologies, Carlsbad, CA, USA), at 37°C in a humidified atmosphere with 5% CO_2_.

Semiconfluent HUVEC cultures (2.5 × 10^5^ cells/well) were stimulated with either vehicle or the human recombinant Dkk-3 peptide (hrDkk-3, R&D Systems) at a final concentration of either 1 or 10 ng/ml.

These concentrations were chosen based on the literature data supporting Dkk-3 ability to induce angiogenesis *in vitro* in primary endothelial colony-forming cells (Untergasser et al., 2008).

Control plates were maintained in *EGM-2* medium. The incubation was stopped after short times (30, 60, and 90 min) and cell lysates were used for western blot analysis of phosphoSmad1/5/8 expression. Longer times incubation (6 and 12 h) were performed for RTPCR, western blot, immunocytochemical analysis of VEGF expression, western blot analysis of the phosphorylated (activated) form of the VEGFR2 receptor. The culture medium was collected for ELISA of the VEGF extracellular levels.

Moreover, HUVECs were tested for endothelial cell tubes formation on Matrigel following different times of incubation (from 1 to 48 h) with hrDkk-3 (10 ng/ml). The endothelial cell tubes formation was evaluated at the end of 18 h of incubation with hrDkk-3 (10 ng/ml) either in presence or in the absence of the selective VEGFR2 antagonist ZM 323881 (1 μM, Tocris Bioscience).

In separate sets of experiments, HUVECs were subjected to ALK1 gene silencing by using small interfering RNAs (siRNA, Sigma Aldrich; Oligo: SASI_Hs01_00113768; Sequence: 5′-CCCUCUACGACUUUCUGCA-3′). After 48 h of transfection, both control and silenced HUVECs were incubated with vehicle or hrDkk-3 (10 ng/ml) and used as follows: (i) cell lysates collected after 1 h of incubation were used for western blot analysis of phosphoSmad1/5/8 expression; (ii) cell lysates collected after 12 h of incubation were used to assess VEGF expression by Western blot and immunocytochemical analysis. Moreover, the endothelial cell tubes formation assay was performed in ALK1 silenced and not silenced HUVECs grown for 18 h on Matrigel either in the presence or in the absence of the Dkk3 peptide (10 ng/ml).

To better assess the role of ALK1 in Dkk-3 mediated VEGF upregulation, the VEGF intracellular expression was assessed by western blot in HUVECs following incubation with hrDkk-3 (10 ng/ml, 12 h) either in the presence or in the absence of the selective ALK1 antagonist ML347 (0.5 or 1 μM, Tocris Bioscience).

Finally, the phospho-Smad1,5,8 expression was evaluated following incubation with hrDkk-3 (10 ng/ml) for 60 min either in the presence or in the absence of neutralizing TGF-β antibody (10 ng/ml, Sigma Aldrich, code: T0438, lot number: 077K1267). The VEGF intracellular expression was assessed after 12 h of incubation with hrDkk-3 in the same experimental condition.

### RTPCR analysis

Total RNA was extracted from cells using Trizol reagent (Life Technologies), subjected to DNAse I treatment (Qiagen, Venlo, Netherlands), and subsequently purified using RNeasy Mini Kit (Qiagen) according to the manufacturer's instructions. RNA integrity was assessed by denaturing agarose gel electrophoresis and its concentration was verified by using NanoDrop2000c UV-Vis spectrophotometer (Thermo Scientific, Waltham, MA, USA).

### VEGF mRNA analysis

One μg of total RNA was used for cDNA synthesis using Superscript VILO master mix (Life Technologies) according to manufacturer's instructions. The following primers were used: VEGF: forward 5′-TCTTCAAGCCATCCTGTGTG-3′; reverse 5′- CTCATCTCTCCTATGTGCTG-3′; GAPDH: forward 5′-ACAGTCAGCCGCATCTTC-3′; reverse 5′-GCCCAATACGACCAAATCC-3′. RTPCR was performed using a 2× SYBR Green PCR Master Mix (Applied Biosystems, Forster City, CA, USA) containing the double-stranded DNA-binding fluorescent probe, Sybr Green, and all necessary components except primers. Quantitative PCR conditions included an initial denaturation step of 94°C/10 min followed by 40 cycles of 94°C/15 s and 60°C/15 s. Measurements were performed in triplicate in each assay. Results were expressed as relative levels of VEGF mRNA at 6 and 12 h compared with basal time.

### ALK1 gene silencing validation

HUVECs subjected to 48 h of transfection with control- or ALK1 silencing siRNA were used for total RNA extraction and cDNA synthesis. RTPCR was used to verify the efficiency of gene silencing by using the following primers: ALK1: forward 5′-ATGACCTCCCGCAACTCGA-3′; reverse 5′- TAGAGGGAGCCGTGCTCGT-3′; β-actin: forward 5′-GCAAGAGATGGCCACGGCTG-3′; reverse 5′-CCACAGGACTCCATGCCCAG-3′. Measurements were performed in triplicate. Results were expressed as relative levels of ALK1 mRNA compared with control cells.

### ChIP

ChIP assay was performed by standard procedures. Cells were fixed with 1% formaldehyde for 10 min at room temperature with swirling. The crosslinking reaction was stopped by adding glycine to a final concentration of 0.125 M, and the incubation was continued for an additional 5 min. Cells were washed three times with cold PBS, harvested by scraping, pelleted, and resuspended in 1 mL of lysis buffer (5 mM PIPES pH 8.0, 85 mM KCl, 0.5% NP40, 1× protease inhibitor) and nuclei lysis buffer (1% SDS, 10 mM EDTA, 50 mM Tris-HCl pH 8.0, protease inhibitor) and then sonicated on ice using a Covaris S220 ultrasonicator. Following sonication, chromatin fragments of 0.2–0.6 kb in length were obtained. Ten percent of the sonicated lysate was saved and successively used to quantify the total amount of DNA present in different samples before immunoprecipitation (Inputs). The chromatin solution was precleared with salmon sperm DNA/protein A-agarose 50% gel slurry for 1 h at 4°C and immunoprecipitated overnight at 4°C with antibody against Smad4 (Cell Signaling Technology, Danvers, USA) or the respective isotype matched control Ig. After precipitation, the chromatin-antibody complexes were collected using Protein A Sepharose beads and washed. Samples were then eluted with 1% SDS, 100 mM NaHCO_3_ at room temperature for 15 min, reverse cross-linked with NaCl 100 mM at 65°C overnight and treated with proteinase-K. Protein-free DNA was extracted by phenol/chloroform/isoamyl alcohol, precipitated with 100% ethanol, and analyzed by RTPCR using the following specific primers for VEGF promoter: forward GCGTGTCTCTGGACAGAGTTT and reverse AGCCTCAGCCCTTCCACA. RTPCR was performed with 15 μl reactions Universal STBR Green (Bio Rad, Hercules, CA, USA) on an Applied Biosystems Step-One instrument. Thermal cycler conditions were as follows: 10 min at 95°C, 40 cycles of denaturation (15 s at 95°C), and combined annealing/extension (1 min at 60°C). The VEGF promoter amplification was estimated by using the following equation: % (DNA-IP/total input) = 2^∧^[Ct(10% input) − 3.32) − Ct (DNA − IP)] × 100%.

### Immunocytochemistry

Following incubation with hrDkk-3, cells were fixed in 4% paraformaldehyde and incubated overnight with a rabbit polyclonal anti-VEGF (1:300; Abcam, Cambridge, UK, code: ab46154, lot number: GR218557-1) and then for 1 h with a secondary biotinylated anti-rabbit antibody (1:200; Vector Laboratories, Burlingame, CA, USA, code: BA-1000; lot number: ZA0324). 3,3-Diaminobenzidine tetrachloride was used for detection. The immunoreactivity was quantified by densitometric analysis.

### Western blot analysis

Western blot analysis were carried out by using the following primary antibodies: rabbit polyclonal anti-phosphorylated Smad1,5,8 (1:1000, Cell Signaling Technology, code: 9511; lot number:14) rabbit polyclonal anti-Smad1 antibody (1:1000, Cell Signaling Technology, code: 9743; lot number: 2) rabbit polyclonal anti-VEGF antibody (1:1000, Abcam, code: ab46154; lot number: GR218557-1), mouse monoclonal anti-β-actin antibody (1:50000, Sigma Aldrich, code: A5441; lot number: 061M4808), rabbit polyclonal anti-Phospho-VEGF receptor 2 (P-VEGFR2) antibody (1:1000, Cell Signaling Technology, code: 2478; lot number: 13) or rabbit polyclonal VEGFR2 (1:1000, Cell Signaling Technology, code: 2479; lot number: 18). After incubation with primary antibodies, filters were washed and incubated with secondary peroxidase-coupled antibodies anti-rabbit (1:6000, Calbiochem, Milan, Italy, code: 401393; lot number: D00051552) or anti-mouse (1:5000, Santa Cruz Biotechnology, Dallas, TX, USA, code: sc-2005; lot number: H0513). Immunostaining was revealed by enhanced chemiluminescence luminosity (GE Healthcare, Milan, Italy).

### VEGF ELISA

After 6 h of incubation with hrDkk-3 (1 or 10 ng/ml), the culture medium was removed and used for the assessment of the VEGF extracellular level. The latter was measured with a VEGF DuoSet enzyme-linked immunosorbent assay (ELISA; R&D Systems, code: DVE00; lot number: 330898), according to the manufacturer's instructions. The results were expressed as pg/mL.

### Angiogenesis assay on matrix gels

HUVEC tubes formation was studied with Matrigel (BD Biosciences, San Jose, CA, USA), as previously reported (Scarpino et al., 2009). Briefly, growth factor-reduced Matrigel was allowed to polymerize for 1 h at 37°C. Cell tubes formation on Matrigel was performed using HUVECs grown for 18 h in a humidified 37°C, 5% CO_2_ incubator. Separate dishes were exposed or not to hrDkk-3 (10 ng/ml) either in the presence or in the absence of the VEGFR2 inhibitor ZM 3238881. The same experimental design was applied to ALK1 gene silenced cells. At the end of exposure, plates were photographed with a microscope (Zeiss, Jena, Germany) fitted with a digital camera. Quantification was performed by using the Angiogenesis Analyzer for ImageJ software in randomly chosen 10X microscopic fields.

### Statistical analyses

**Figure 1:**
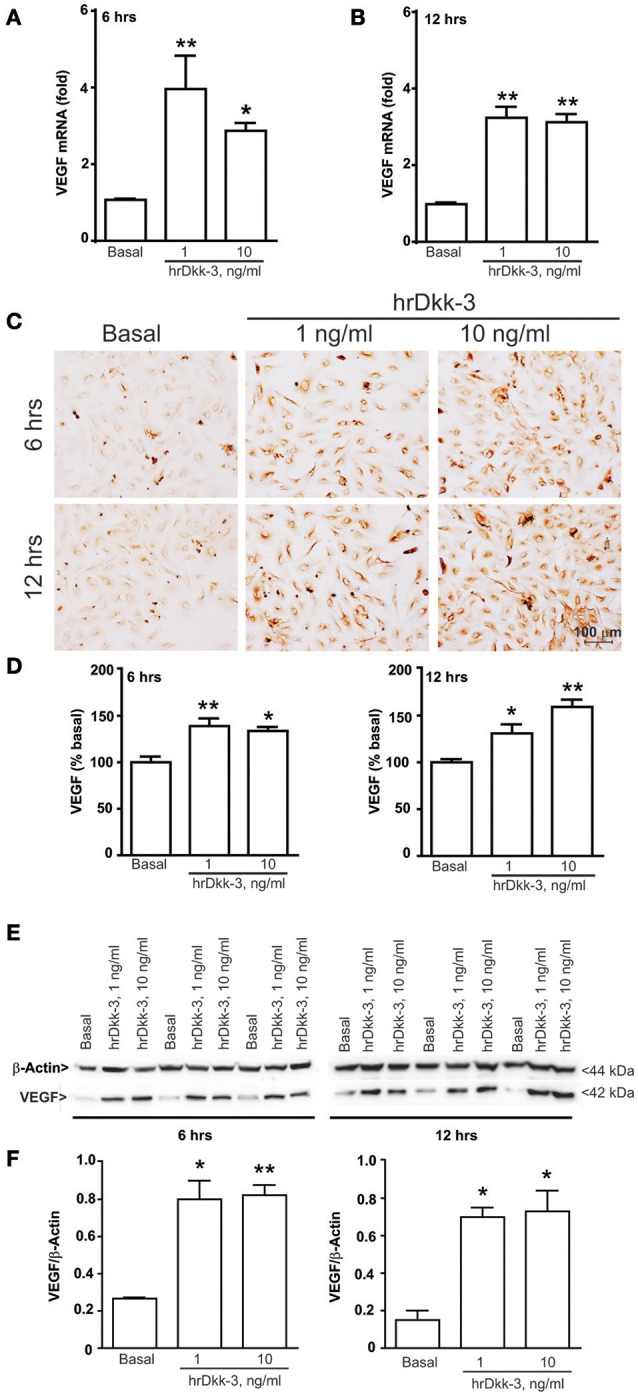
**Dkk-3 upregulates the intracellular VEGF expression level**. RTPCR **(A,B)**, immunocytochemical **(C,D)**, western blot analysis **(E,F)** of VEGF in HUVECs incubated with hrDkk-3 (1 ng/ml or 10 ng/ml) for either 6 or 12 h. Values are means ± SEM. ^*^p < 0.05 vs. baseline condition; ^**^p < 0.001 vs. baseline condition.

**Figure 2:**
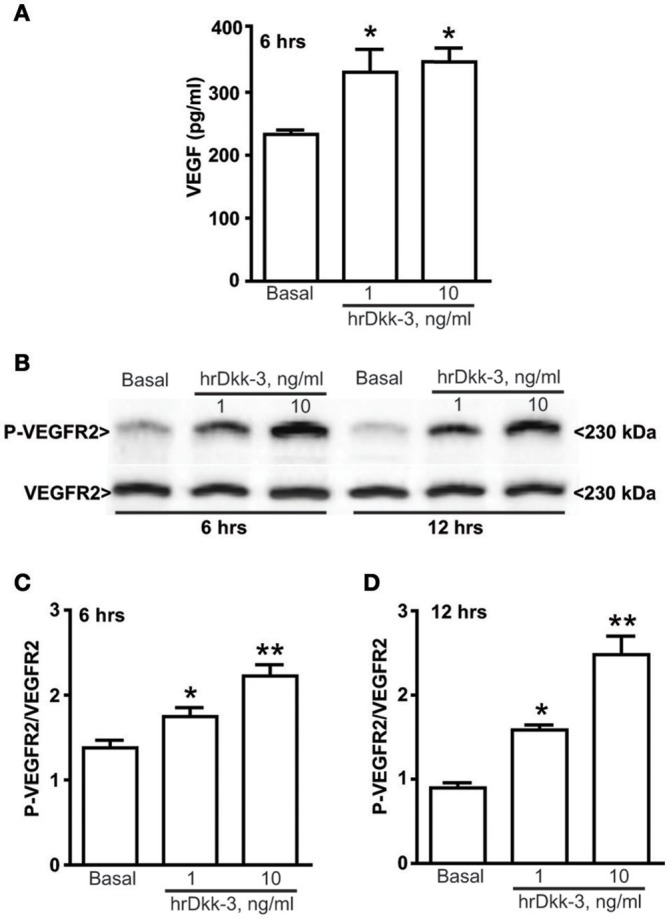
**Dkk-3 upregulates the extracellular VEGF expression level and increases the VEGFR2 activation. (A)** ELISA of VEGF in the medium cell culture after incubation with hrDkk-3 (1 ng/ml or 10 ng/ml) for 6 h. Values are means ± SEM. ^*^p < 0.05 vs. baseline condition. **(B)** Western blot analysis of P-VEGFR2 in cells incubated with hrDkk-3 (1 ng/ml or 10 ng/ml) for either 6 or 12 h. **(C,D)** Corresponding densitometric analysis. Values are means ± SEM. ^*^p < 0.05 vs. baseline condition, ^**^p < 0.001 vs. baseline condition.

**Figure 3:**
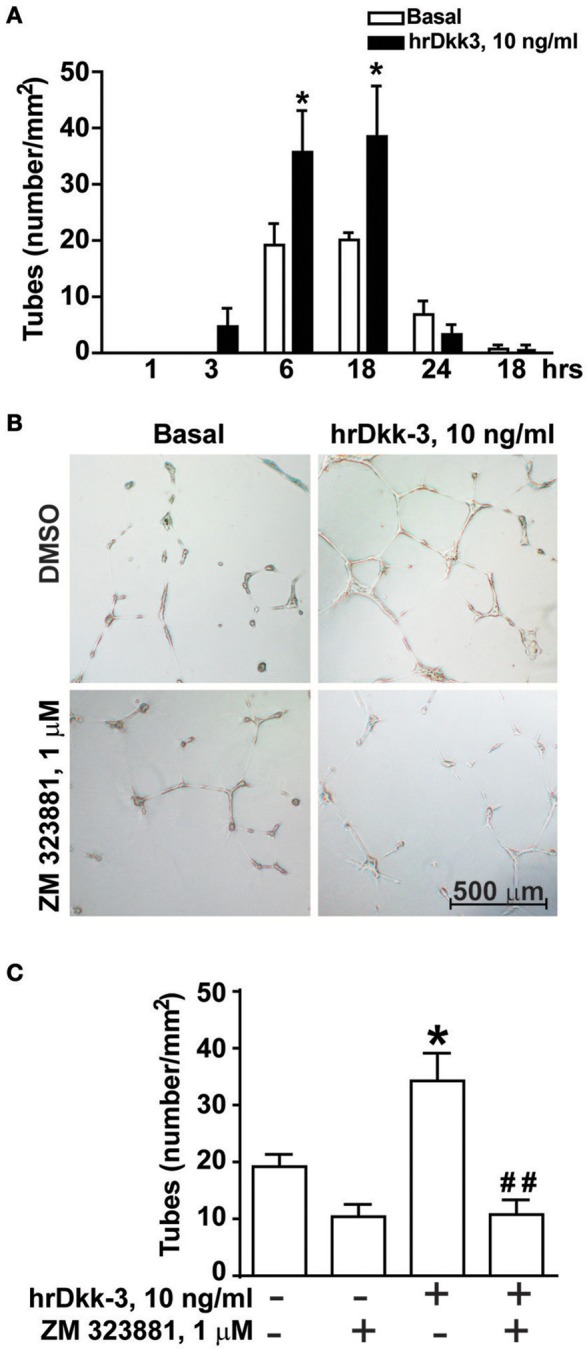
**Angiogenic-like activity of Dkk-3 is mediated by VEGF. (A)** Quantification of endothelial cell tubes on Matrigel at different times of incubation with hrDkk-3. Values are means ± SEM. ^*^p < 0.05 vs. baseline condition. **(B,C)** Endothelial cell tubes formation following 18 h of incubation with hrDkk-3 either in the presence or in the absence of the selective VEGFR2 receptor antagonist ZM 323881. In **(B)** representative images of endothelial cell tube are shown following 18 h of incubation with hrDkk-3 and/or ZM 323881. In **(C)** the corresponding quantification of endothelial cell tubes are shown. Values are means ± SEM. ^*^p < 0.05 vs. baseline condition; ^*##*^*p* < 0.001 vs. hrDkk-3.

**Figure 4:**
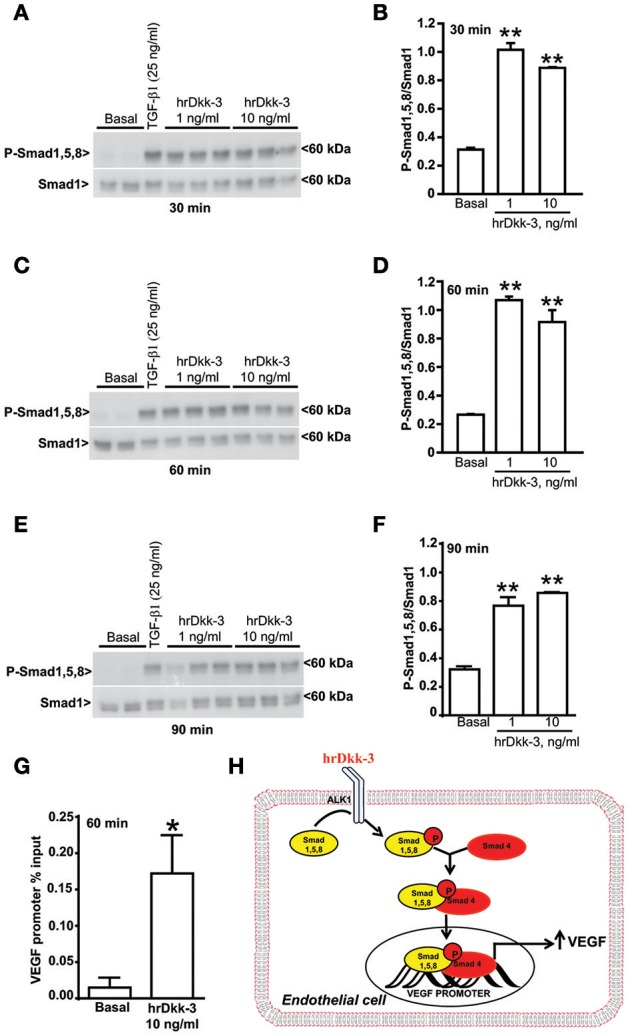
**Dkk-3 enhances the expression of phospho-Smad1/5/8. (A–F)** Western blots and the corresponding densitometric analysis of phospho-Smad1/5/8 in cells incubated with hrDkk-3 for different times (30, 60, and 90 min). HUVECs incubated with TGF-β1 (25 ng/ml) were used as positive controls. Values are means ± SEM. ^**^p < 0.001 vs. baseline condition. **(G)** ChIP assay showing the increased binding of Smad4 to the VEGF promoter in response to the incubation with hrDkk-3 (10 ng/ml, 60 min). Values are means ± SD and are expressed as percentage of inputs. ^*^p < 0.05 vs. baseline condition. **(H)** Schematic representation of the signaling events occurring in cells in response to Dkk-3 exposure and leading to VEGF transcriptional upregulation through a recruitment of the Smad1,5,8 and Smad4.

**Figure 5:**
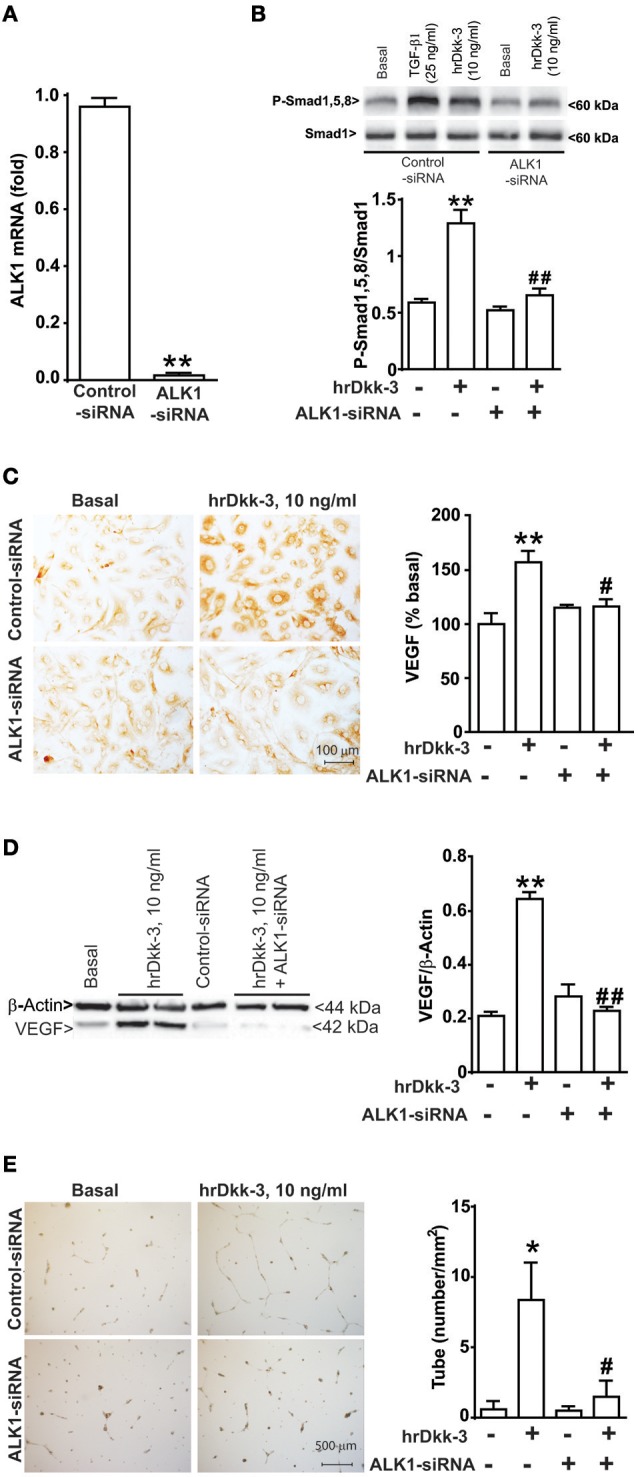
**ALK1 receptor gene silencing by siRNA abrogates Smad1/5/8 phosphorylation, VEGF upregulation and angiogenesis induced by Dkk-3. (A)** RTPCR analysis of ALK1 mRNA level in both control and ALK1 silenced cells. ^**^p < 0.001 vs. control-siRNA treated cells. **(B)** Western blot analysis of phospho-Smad1,5,8 expression in both control and ALK1 silenced cells exposed to hrDkk-3 (10 ng/ml, 60 min). HUVECs incubated with TGF-β1 were used as positive controls. Values are means ± SEM. ^**^p < 0.001 vs. baseline conditions, ^##^*p* < 0.001 vs. hrDkk-3. Immunocytochemical **(C)** and western blot analysis **(D)** of VEGF expression in both control and ALK1 silenced cells exposed to hrDkk-3 (10 ng/ml, 12 h). Values are means ± SEM. ^**^p < 0.001 vs. baseline conditions, ^#^*p* < 0.05 vs. hrDkk-3, ^*##*^*p* < 0.001 vs. hrDkk-3. **(E)** Endothelial cell tube formation of both control and ALK1 silenced cells treated with hrDkk-3 (10 ng/ml, 18 h). Values are means ± SEM. ^*^p < 0.05 vs. baseline conditions, ^#^*p* < 0.05 vs. hrDkk-3.

**Figure 6:**
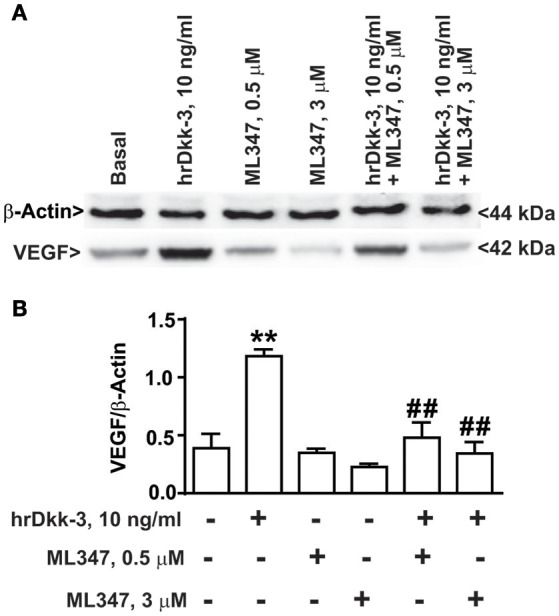
**ALK1 receptor pharmacological blockade abrogates VEGF upregulation induced by Dkk-3 in HUVECs. (A)** Western blot analysis of VEGF expression in HUVECs exposed to hrDkk-3 (10 ng/ml, 12 h) either in the presence or in the absence of the selective inhibitor ML347 (0.5 M or 3 μM). **(B)** Corresponding densitometric analysis. Values are means ± SEM. ^**^p < 0.001 vs. baseline conditions, ^*##*^*p* < 0.001 vs. hrDkk-3.

Statistical analyses were performed using unpaired *t* test (**Figures 4G, 5A**) and One Way ANOVA followed by Bonferroni's LSD test (Figures 1A, **5B**) or Fisher's LSD test (Figures 1D,F, 2C,D, 3C, 4B,D,F, 5C,D, 6B) or Dunnett's LSD test (Figure 2A) or Tukey's LSD test (Figure 5E) and Two Way ANOVA followed by Fisher (Figure 3A).

## Results

### VEGF was upregulated in HUVECs following incubation with hrDkk-3

To evaluate whether Dkk-3 modulates VEGF expression in the endothelium, we performed *in vitro* experiments using cultured HUVECs. Treatment with hrDkk-3 resulted in a marked upregulation of mRNA VEGF level (Figures 1A,B).

Immunocytochemical and western blot analyses showed a significant upregulation of the VEGF intracellular protein level following incubation with hrDkk-3 at both 6 and 12 h (Figures 1C–F). Moreover, a large increase in the extracellular VEGF level was observed following incubation with hrDkk-3 (1 or 10 ng/ml) (Figure 2A). This increase was associated to an enhanced VEGFR2 phosphorylation (Figures 2B,C).

### Dkk-3-induced angiogenesis is mediated by VEGF

The proangiogenic activity of hrDkk-3 was demonstrated by a significant increase in the time course of endothelial cell tubes formation at both 6 and 18 h (Figure 3A).

To examine whether VEGF was involved in the angiogenic effect of Dkk-3, we repeated the endothelial cell tubes formation assay in the presence of the pharmacological blockade of VEGR2. In fact, ZM 323881, which was inactive on its own, abrogated the enhancing effect of Dkk-3 on angiogenesis (Figures 3B,C).

### Dkk-3 enhanced Smad1,5,8 phosphorylation

We studied the mechanism by which hrDkk-3 enhanced VEGF expression and promoted *in vitro* angiogenesis focusing on TGF-β receptor signaling. Moving from the evidence that TGF-β stimulates endothelial cell proliferation *via* type-1 TGF-β/ALK1 receptor activation and the ensuing phosphorylation of Smad1,5,8 transcription factors (Lebrin et al., 2005), we examined whether this signaling mechanism could be involved in the molecular actions of Dkk-3 in HUVECs. The western blot analysis showed a significant increase in phosphorylated-Smad1,5,8 levels in HUVECs treated with hrDkk-3 at all incubation times (Figure 4). These data suggest that Dkk-3 is able to activate, either directly or indirectly, ALK1 receptor.

Finally, we performed a ChIP analysis in order to evaluate whether the exposure to Dkk-3 may lead to an increased Smad4 binding to the VEGF promoter. Our results showed an increased binding of Smad4 to the VEGF promoter in response to incubation with Dkk-3 (Figure 4G). Therefore, the molecular mechanism of Dkk-3-mediated VEGF upregulation appears to be a transcriptional activation that occurs through the Smad1,5,8 phosphorylation and the ensuing recruitment of the Smad4 factor (Figure 4H).

### ALK1 gene silencing inhibited Smad1,5,8 phosphorylation, the enhanced VEGF expression and endothelial cell tubes formation induced by Dkk-3

To examine whether ALK1 activation was causally related to the effect of Dkk-3 on Smad1,5,8 phosphorylation, VEGF expression and angiogenesis, we silenced ALK1 gene with a specific siRNA. The efficiency of ALK1 gene silencing was verified by RTPCR for 48 h after transfection (Figure 5A).

The ALK1 gene silencing prevented the increase in phosphorylated-Smad1,5,8 levels induced by hrDkk-3 (Figure 5B). Moreover, ALK1 silencing abrogated the VEGF upregulation and the angiogenesis process induced by hrDkk-3 (Figures 5C–E).

These data demonstrate that ALK1 activation lies upstream in the chain of events leading to VEGF induction and angiogenesis in response to Dkk-3.

### ALK1 pharmacological blockade inhibited the VEGF upregulation induced by Dkk-3

In order to confirm the involvement of the ALK1 receptor in hrDkk-3 mediated VEGF upregulation, we examined the effect of ALK1 pharmacological blockade by using the selective ALK1 inhibitor ML347. Our results showed that ML347 exposure abrogated the VEGF upregulation induced by hrDkk-3 (Figure 6).

### TGF-β neutralization does not inhibit Smad1,5,8 phosphorylation and the VEGF upregulation induced by Dkk-3

**Figure 7:**
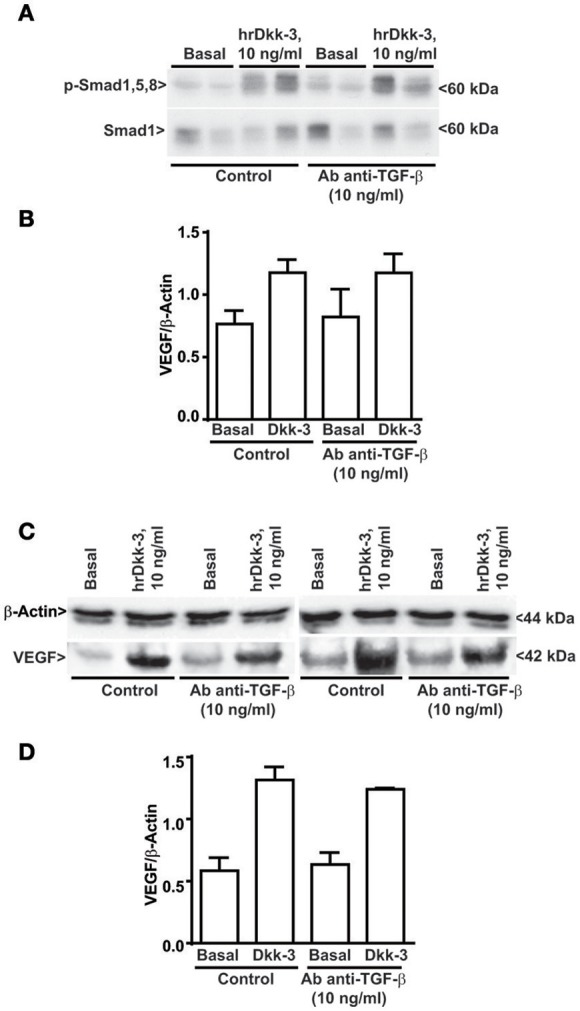
**TGF-β neutralizing antibody does not inhibit Smad1,5,8 phosphorylation and the VEGF upregulation induced by Dkk-3. (A)** Western blot analysis of phosphoSmad1,5,8 in cells exposed for 60 min to hrDkk-3 either in the presence or in the absence of neutralizing TGF-β antibody. **(B)** Corresponding densitometric analysis. Values are means ± SD. **(C)** Western blot analysis of VEGF in cells exposed for 12 h to hrDkk-3 either in the presence or in the absence of neutralizing TGF-β antibody. **(D)** Corresponding densitometric analysis. Values are means ± SD.

We used a specific TGF-β neutralizing antibody to evaluate whether the ability of Dkk-3 to activate the ALK1 signaling and stimulate VEGF upregulation occurs through a cross talk with TGF-β. Our results showed that the TGF-β neutralization does not interfere with Dkk-3-mediated Smad1,5,8 phosphorylation (Figures 7A,B) or with VEGF upregulation (Figures 7C,D). These data suggest that the molecular axis Dkk-3/ALK1/VEGF does not require a cross talk between Dkk-3 and TGF-β.

## Discussion

Dkk-3 was initially identified as a tumor suppressor (Tsuji et al., 2000, 2001), and was also shown to be involved in epithelial cell senescence (Untergasser et al., 2002), and acinar cell differentiation and morphogenesis (Kawano et al., 2006). More recent studies have demonstrated that Dkk-3 acts as a differentiation factor involved in remodeling of tumor vasculature (Untergasser et al., 2008). In particular, Dkk-3 is strongly expressed in tumor endothelial cells, and appears to be involved in capillary formation and angiogenesis (Untergasser et al., 2008).

Thus, Dkk-3 may contribute to maintain blood vessel wall structure both under physiological conditions and in response to pathological stimuli through its ability to act as an angiogenic factor.

The angiogenic activity of Dkk-3 was demonstrated in cultures of endothelial colony-forming cultures as shown by the altered endothelial cell tubes formation on Matrigel after Dkk-3 gene downregulation or adenoviral overexpression (Untergasser et al., 2008).

The molecular mechanisms underlying the angiogenic action of Dkk-3 at the endothelial level have not been yet identified.

Herein, we have shown for the first time that Dkk-3 increases the intracellular VEGF expression and the extracellular VEGF release with an ensuing activation of VEGFR2 receptor in endothelial cells. Moreover, we demonstrated that activation of VEGFR2 was ultimately responsible for the *in vitro* angiogenic-like effects of Dkk-3.

VEGF plays a fundamental role in mechanisms of vasculogenesis and angiogenesis in both physiological and pathological conditions (Ferrara et al., 2003; Ferrara, 2004). VEGF acts by inducing endothelial cell proliferation, survival and migration resulting into the formation of capillary-like tubules (Darland et al., 2003; Gerhardt et al., 2003). Genetic deletion of a single *VEGF* allele leads to embryonic death with severe vascular defects (Carmeliet et al., 1996; Ferrara et al., 1996). In addition, VEGF has been implicated in the physiological control of vasculature function as well as in vascular dysfunction associated with neural grafting, tumor cell implantation, tumor development, transient forebrain ischemia, and hypertension (Krum and Rosenstein, 1998; Nishikawa et al., 1998; Lee et al., 1999; Chow et al., 2001; Ferrara et al., 2004; Hurwitz et al., 2004; Hicklin and Ellis, 2005; Bhargava, 2009). Therefore, the functional link between Dkk-3 and VEGF currently identified in endothelial cells raises the possibility that Dkk-3 represents an important molecular player involved in vascular health and disease through VEGF modulation.

We performed experiments to characterize the molecular mechanism underlying the VEGF upregulation and the related angiogenic activity dependent on Dkk-3 in HUVECs. The main difficulty was related to the lack of information of a putative Dkk-3 receptor. Unlike the other Dkk members, Dkk3 does not interact with the Wnt signaling (Krupnik et al., 1999; Wu et al., 2000; Mao B. et al., 2001; Mao J. et al., 2001) and it does not show affinity to the Wnt co-receptors LRP5/6 and Kremen (Mao B. et al., 2001; Mao J. et al., 2001; Mao et al., 2002; Diep et al., 2004).

Remarkably, Dkk-3 is able to up-regulate ATF3 and enhance Smad3 phosphorylation in tumor cell lines (Kashiwakura et al., 2008). Moreover, depletion of Dkk-3 in Xenopus embryos by antisense oligonucleotides strongly reduced levels of Smad4 protein (Pinho and Niehrs, 2007). Both ATF3 and Smads are components of the intracellular signaling pathway activated by TGF-β.

We therefore focused on TGF-β signaling as a potential mediator of Dkk-3 actions in endothelial cells.

TGF-β has an established role in vascular homeostasis (Ten Dijke and Arthur, 2007) and angiogenesis (Pepper, 1997; Goumans et al., 2003). In fact, mice lacking TGF-β1 die *in utero* owing to vascular defects (Dickson et al., 1995). TGF-β activates type-I and type-II transmembrane serine/threonine-kinase receptors. Binding of TGF-β to the type-II receptor leads to recruitment and phosphorylation of type-I receptor ALK1 and ALK5. Following activation of type I receptors, the signal is transduced from the membrane to the nucleus via intracellular effectors, called Smads (Derynck and Zhang, 2003; Ten Dijke and Hill, 2004). Activated ALK1 phosphorylates the downstream effectors, Smad1, Smad5, and Smad8, whereas activated ALK5 phosphorylates Smad2 and Smad3 (Goumans et al., 2002).

TGF-β modulates endothelial cell proliferation and migration *via* a fine balance between ALK5 and ALK1 signaling, with ALK1 enhancing endothelial cell migration and proliferation, and ALK5 doing the opposite (Goumans et al., 2002; Lebrin et al., 2005). TGF-β can also modulate angiogenesis by regulating the expression of VEGF in vascular endothelial cells (Seghezzi et al., 1998; Ferrari et al., 2006).

Here, we have shown that Dkk-3 was able to activate ALK1 receptor in endothelial cells, as suggested by the increase in Smad1/5/8 phosphorylation, and to induce the Smad4 recruitment to the VEGF promoter. The ALK1 receptor activation laid upstream in the chain of events induced by Dkk-3, since both ALK1 silencing and the pharmacological blockade of ALK1 receptor abrogated the Dkk-3-induced VEGF expression and angiogenesis. These data demonstrate that the type-I TGF-β/ALK1 receptor plays a key role in mediating the action of Dkk-3 in endothelial cells.

Moreover, experiments of TGF-β neutralization allowed us to demonstrate that the Dkk-3 mediated activation of ALK1 receptor does not require a cross talk between Dkk-3 and TGF-β. We cannot exclude the existence of a molecular partner of Dkk-3 favoring its action on ALK1 receptor. On the other hand, the presence of TGF-β is not required for ALK1 activation and VEGF upregulation mediated by Dkk-3 in endothelial cells. Therefore, whether Dkk-3 is a direct activator of TGF-β receptor or it acts on a different membrane receptor that trans-activates ALK1 remains to be determined.

Taken together our data demonstrate that Dkk-3 acts on endothelial cells by activating the ALK1 receptor with ensuing Smad1,5,8 phosphorylation, Smad4 recruitment to the VEGF promoter, transcriptional upregulation of VEGF which, in turn, mediates the angiogenic activity through the VEGFR2 activation.

The functional link between Dkk-3 and ALK1 in endothelial cells suggests a role for Dkk-3 in vascular pathology. Smad1 and Smad5 are phosphorylated in endothelial cells in response to oscillatory shear stress resulting into cell cycle progression (Zhou et al., 2012). Thus, Dkk-3 might promote vascular remodeling under pathological conditions by enhancing Smad1, Smad5 and Smad8 phosphorylation. According to this hypothesis, mutations in the ALK1 receptor gene have been associated with vascular disorders, such as Haemorrhagic Telengiectasia (Johnson et al., 1996) and pulmonary arterial hypertension (Machado et al., 2009; Jerkic et al., 2011; Gore et al., 2014). In addition, heterozygous disruption of ALK1 receptor is associated with increased arterial blood pressure in mice (González-Núñez et al., 2015).

## Conclusions

We have shown for the first time the following cascade of events in endothelial cells:

1. Dkk-3 activates type-I TGF-β receptor, ALK1, inducing phosphorylation of Smad1/5/8 and the Smad4 recruitment to the VEGF promoter;
2. Dkk-3-mediated ALK1 activation leads to a VEGF transcriptional upregulation;
3. Activation of VEGF receptor mediates the angiogenic activity of Dkk-3.

The functional characterization of Dkk-3/ALK1/VEGF as a new molecular axis in the endothelial vascular compartment highlights a novel role of Dkk-3, raising the possibility that the chain of events triggered by Dkk-3 might represent a novel target for the treatment of vascular diseases.

## Author contributions

CB designed and performed experiments, analyzed data and wrote the manuscript. SM and FB designed and performed experiments. PDP analyzed formation of capillary tubes for angiogenesis. BR performed Western blot analysis. RS validated siRNA for *in vitro* experiments. MC performed ChIP analysis. GB, VB, MV, FF, FN, and SR conceived the study, designed, interpreted experiments and wrote the manuscript.

## Funding

This work was supported by the Italian Ministry of Health (Project code: GR-2011-02350132, 2011) and by the 5‰ grant to MV and SR.

### Conflict of interest statement

The authors declare that the research was conducted in the absence of any commercial or financial relationships that could be construed as a potential conflict of interest.

## Abbreviations

ALK: Activin Like Kinase
ChIP: Chromatin Immunoprecipitation
Dkk-3: Dickkopf-3
EDTA: Ethylenediaminetetraacetic Acid
*EGM-2*: Endothelial Cell Growth Medium-2
ELISA: Enzyme-Linked Immunosorbent Assay
HUVECs: Human Umbilical Vein Endothelial Cells
hrDkk-3: human recombinant Dkk-3
PBS: Phosphate Buffer Solution
PIPES: Piperazine-N,N′-bis(Ethanesulfonic acid)
RTPCR: Real Time Polymerase chain reaction
SDS: Sodium dodecyl sulfate
SiRNA: Short interfering RNA
Smad: small mother against decapentaplegic
TGF-β: Transforming Growth Factor beta
Tris-HCl: Trisaminomethane Hydrochloride
VEGF: Vascular Endothelial Growth Factor
VEGFR2: Vascular Endothelial Growth Factor Receptor 2
Wnt: Wingless Integration site.
